# 

**Published:** 1848-01

**Authors:** 


### LIBRARY.

PAGE.

Complete Elements of the Science and Art of the Dentist. Translated from the French of M. Desirabode, by *—A—*, . . . . 497

An Essay on the Structure and Formation of the Teeth in Man and Various Animals. By Robert Blake, M. D., Revised and Corrected with Notes, by C. O. Cone, D. D. S. . . . . 1

### JOURNAL.

Article I.—Observations upon the Importance and Value of a Knowledge of the Collateral Branches of Medicine and Surgery in connection with Dentistry. By James Robinson, D. D. S. London, . . . . . . . . . . . . . 113
Article II.—A Course of Lectures on Dental Physiology and Surgery, delivered at the Middlesex Hospital School. By John Tomes, Esq., Surgeon Dentist to the Hospital, . . . . 120
Article III.—Account of a New Anæsthetic Agent, as a Substitute for Sulphuric Ether in Surgery and Midwifery. By J. Y. Simpson, M. D., F. R. S. E. . . . . . . . . . . 147
Article IV.—Letter from Dr. S. Parsons, . . . . . . 159
Article V.—Letter from Dr. G. F. J. Colburn, . . . . . 160
Article VI.—Physical Diagnosis in Determining the Amount of Muscular Effort Demanded in the Operation for the extraction of Teeth. By C. O. Cone, D. D. S., Baltimore, . . . . 162
Article VII.—Letter from Dr. S. C. Harbert, . . . . . 170
Article VIII.—Letter from Prefessor Slack, to Professor James Taylor, M. D., . . . . . . . . . . . . . 172
Article IX.—Effects of Camphor on the Teeth, . . . . . 178
Article X.—Premature Dentition and Supernumerary Teeth, . 181
Article XI.—On Cleft Palate, and on Staphyloraphy. By William Furgusson, F. R. S. E. . . . . . . . . . . 184

### MISCELLANEOUS NOTICES.

Correction, . . . . . . . . . . . . . . . . 192

Chloroform, a New Anæsthetic Agent, . . . . . . . 193

Mississippi Valley Association of Dental Surgeons, . . . . 198

Nævus, . . . . . . . . . . . . . . . . 199

Amalgam, . . . . . . . . . . . . . . . 199

New York Dental Recorder, . . . . . . . . . . 203

The Dental Register of the West, . . . . . . . . 203

Fourth Dentition, . . . . . . . . . . . . . 204

The Dental News Letter, . . . . . . . . . . . 206

Obstinate Hæmorrhage from the Extraction of Teeth, . . . . 207

A New Wax-holder, for taking Impressions of the Mouth, . . . 208

A New Antagonizing Instrument for double sets of Artificial Teeth, . 208

Correction, . . . . . . . . . . . . . . . 208

